# Phosphatidylcholine induces apoptosis of 3T3-L1 adipocytes

**DOI:** 10.1186/1423-0127-18-91

**Published:** 2011-12-07

**Authors:** Hailan Li, Jong-Hyuk Lee, Su Yeon Kim, Hye-Young Yun, Kwang Jin Baek, Nyoun Soo Kwon, Yoosik Yoon, Ji Hoon Jeong, Dong-Seok Kim

**Affiliations:** 1 Departments of Biochemistry, Chung-Ang University College of Medicine, 221 Heukseok-dong, Dongjak-gu, Seoul 156-756, Republic of Korea; 2 Departments of Pharmacology, Chung-Ang University College of Medicine, 221 Heukseok-dong, Dongjak-gu, Seoul 156-756, Republic of Korea; 3 Departments of Microbiology, Chung-Ang University College of Medicine, 221 Heukseok-dong, Dongjak-gu, Seoul 156-756, Republic of Korea

**Keywords:** adipocytes, apoptosis, caspases, mesotherapy, PPC

## Abstract

**Background:**

Phosphatidylcholine (PPC) formulation is used for lipolytic injection, even though its mechanism of action is not well understood.

**Methods:**

The viability of 3T3-L1 pre-adipocytes and differentiated 3T3-L1 cells was measured after treatment of PPC alone, its vehicle sodium deoxycholate (SD), and a PPC formulation. Western blot analysis was performed to examine PPC-induced signaling pathways.

**Results:**

PPC, SD, and PPC formulation significantly decreased 3T3-L1 cell viability in a concentration-dependent manner. PPC alone was not cytotoxic to CCD-25Sk human fibroblasts at concentrations <1 mg/ml, whereas SD and PPC formulation were cytotoxic. Western blot analysis demonstrated that PPC alone led to the phosphorylation of the stress signaling proteins, such as p38 mitogen-activated protein kinase and c-Jun N-terminal kinase, and activated caspase-9, -8, -3 as well as cleavage of poly(ADP-ribose) polymerase. However, SD did not activate the apoptotic pathways. Instead, SD and PPC formulation induced cell membrane lysis, which may lead to necrosis of cells.

**Conclusions:**

PPC results in apoptosis of 3T3-L1 cells.

## Background

Mesotherapy is a new technique of injecting drugs into the mesoderm for the treatment of local conditions [1]. This method allows the use of increased concentration of drugs and can produce greater treatment effects. Mesotherapy has been used for many infirmities, such as fat emboli, chronic pain, hyperlipidemia, and liver problems [2]. Phosphatidycholine (PPC) formulation has been widely-used to dissolve local fat deposits as a safe nonsurgical alternative to liposuction [3]. Many clinical studies have reported that the subcutaneous injection of PPC formulation reduces fat [3-5]. Although the biochemical mechanisms are very poorly studied, it has been suggested that no enzymatic lipolytic pathway is involved [6]. Thus, it is thought that the PPC formulation dissolves local fat deposits in a nonspecific manner [7]. PPC formulation has not been approved by the Food and Drug Administration (FDA) for use in lipodissolution.

PPC is a lecithin-derived phospholipid [4], which is widely-distributed in human cell membranes [8]. The Lands cycle and the Kennedy pathway are two pathways of PPC synthesis [9], and they take place in the endoplasmic reticulum [10]. Increased PPC in cell membranes can accelerate lipolysis by improving sensitivity to insulin [5,11]. In addition, PPC is the major phospholipid in pulmonary surfactant [12]. On the other hand, PPC induces apoptosis of hepatic cancer cells [13]. Moreover, the size of lipomas is reduced after intralesional injection of PPC [14].

Obesity is one of the main health problems in much of the Western world, as a consequence of the induction of various metabolic derangements including dyslipidemia, hypertension, glucose intolerance, and hepatic steatosis [15]. Obesity is identified by increased number and size of adipocytes [16]. Adipocytes, which store excess energy, release paracrine factors that induce growth and differentiation of neighboring pre-adipocytes [17]. Therefore, both suppression of pre-adipocyte differentiation and decrease in cell viability of pre-adipocytes and adipocytes are possible ways to treat obesity. To date, many studies have focused on the inhibition of adipogenesis. However, the notable ability of adipocytes to resist apoptosis is poorly understood [18].

Cell death, including apoptosis and necrosis, are followed by the cleavage of proteins and DNA [19]. These two pathways of cell death are associated with different patterns of nuclear protein cleavage [20]. For example, poly(ADP-ribose) polymerase (PARP) cleavage generates an 85 kD fragment during apoptosis but generates a 50 kD fragment in necrotic cell death [21]. Caspase-3 is an apoptotic signal transducer in 3T3-L1 pre-adipocytes [22].

In the present study, PPC formulations containing PPC and sodium deoxycholate (SD) were used. SD is a secondary bile salt and is used as a laboratory detergent to dissolve PPC [7]. Physiologically, however, SD, not PPC, could be the major active component for fat lysis [7,23]. To address this speculation, the present study assessed the effects of PPC alone, PPC formulation, and SD on 3T3-L1 pre-adipocytes and differentiated 3T3-L1 adipocytes. Until now, the actions of PPC and SD could not be directly compared, because PPC needs to be emulsified with SD. However, we could apply PPC alone using bovine serum albumin (BSA) as a carrier.

The specific aim of this study was to investigate the mechanism of cell death induced by PPC and/or SD in 3T3-L1 pre-adipocytes. Furthermore, our study focused on the apoptotic effect of PPC on 3T3-L1 pre-adipocytes.

## Materials and methods

### Materials

L-α-phosphatidylcholine from soybean (P7443, ≥99%), fatty acid-free BSA, insulin, dexamethasone, 3-isobutyl-1-methylxanthine, and 3-(4, 5-dimethylthiazol-2-yl)-2, 5-diphenyltetrazolium bromide (MTT) were obtained from Sigma-Aldrich (St. Louis, MO, USA). PPC was added to cells as a complex with 0.4% BSA. Antibodies against phospho-p38 mitogen-activated protein kinase (MAPK) (Thr180/Tyr182) (CST-9211), phospho-c-Jun-N-terminal kinase (JNK) (Thr183/Tyr185) (CST-9251), and cleaved caspase-3 (CST-9661) were obtained from Cell Signaling Technology (Danvers, MA, USA). Antibodies against Bax (sc-526), Bcl-2 (sc-7382), caspase-9 (sc-8355), caspase-8 (sc-7890), PARP (sc-7150), and actin (sc-1616) were purchased from Santa Cruz Biotechnology (Santa Cruz, CA, USA). SD was obtained from New Zealand Pharmaceuticals (Palmerston, New Zealand) and a type of PPC formulation, Lipobean^® ^(50 mg/ml, containing 2.4% SD) was kindly supplied by AmiPharm (Seoul, Korea).

### Cell cultures

3T3-L1 pre-adipocytes were obtained from ATCC (Rockville, MD, USA). The cells were grown in Dulbecco's Modified Eagle's Medium (DMEM) supplemented with 10% bovine calf serum (BCS; Invitrogen, Carlsbad, CA, USA), 50 μg/ml of streptomycin, and 50 μg/ml of penicillin at 37°C in 5% CO_2_. After cells were reached confluence, designated day 0, the cells were maintained with differentiation-induction medium containing 0.5 mM 3-isobutyl-1-methylxanthine, 0.25 μM dexamethasone, 1 μg/ml insulin (MDI) and in DMEM with 10% BCS for 4 days. Next, the culture medium was changed to differentiation maintenance medium containing 1 μg/ml insulin in DMEM with 10% BCS for 2 days.

### MTT assay for cell viability

Cells (1 × 10^4 ^cells/well), seeded into 12-well plates for 24 h, were incubated with various concentrations of PPC, SD, and Lipobean^® ^(0-1 mg/ml) in DMEM containing 10% fetal bovine serum (FBS) at 37°C in 5% CO_2 _for 24 h. After adding 100 μl/well of MTT solution (5 mg/ml), the plates were incubated for another 4 h. Supernatants were then removed and the formazan crystals were solubilized in 1 ml of dimethylsulfoxide. Optical density was determined at 540 nm using a VERSAMax ELISA reader (Molecular Devices, Sunnyvale, CA, USA).

### Cell cycle analysis

Cells were trypsinized, adjusted to a density of 5 × 10^5 ^- 1 × 10^6 ^cells/tube, washed with ice-cold phosphate-buffered saline (PBS), and re-suspended in 2 ml of ethanol. After incubation at 4°C for 1 h, the ethanol was removed and 100 μl of ribonuclease solution (10 mg/ml) was added to each test tube. The tubes were then re-incubated at room temperature for 30 min, and 500 µl of analysis solution (37 mM EDTA and 0.1% Triton X-100 in PBS) and 100 μl of propidium iodide solution (400 μg/ml) were then added. Samples were stored in the dark at 4°C and analyzed using a FACSCalibur flow cytometer (Becton Dickinson, San Jose, CA, USA).

### Western blotting

Cells were grown in 100 mm-diameter culture dishes, serum-starved for 24 h, and treated with PPC, SD, and Lipobean^® ^at the indicated times. Cell lysates were prepared in M-PER mammalian protein reagent (Pierce, Rockford, IL, USA) containing a complete protease inhibitor mixture (Roche, Mannheim, Germany). Samples were separated by 12% sodium dodecyl sulfate-polyacrylamide gel electrophoresis (SDS-PAGE) and the resolved proteins were transferred to polyvinylidene fluoride (PVDF) membranes, which were blocked with 5% dried milk in PBS containing 0.5% Tween 20. The blots were incubated with the appropriate primary antibodies at a dilution of 1:1000. Membrane-bound primary antibodies were detected using secondary antibodies conjugated with horseradish peroxidase and chemiluminescent substrate (Pierce). The images of the blotted membranes were obtained using a LAS-1000 lumino-image analyzer (Fuji Film, Tokyo, Japan).

### Statistical analyses

Differences between groups were assessed with an analysis of variance, followed by the Student's *t*-test. *P *values <0.01 were considered significant.

## Results

### Effects of PPC, SD, and Lipobean^® ^on 3T3-L1 pre-adipocyte viability

**Figure 1:**
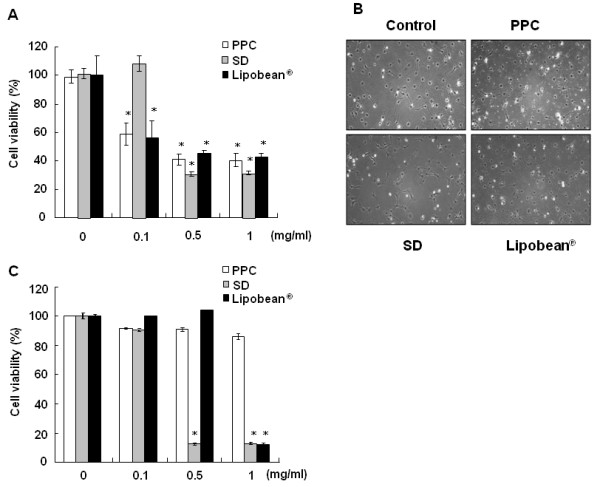
**Effects of PPC on cell viabilities**. (A) 3TC-L1 cells were treated with PPC, SD, Lipobean^® ^at 0-1 mg/ml. (B) Morphology of 3T3-L1 cells after 0.1 mg/ml of PPC, SD, Lipobean^® ^treatment. Phase contrast photographs were taken using a digital video camera. (C) CCD-25Sk human fibroblasts were treated with PPC, SD, Lipobean^® ^at 0-1 mg/ml. After 24 h, cell viability was measured by MTT assay. The data represent the mean ± standard deviation (S.D.) of triplicate assays expressed as percentages of the control. Each experiment was repeated independently at least twice, and the representative results are shown. * *P *< 0.01 compared to the untreated control.

To examine cytotoxicity, 3T3-L1 pre-adipocytes were treated with PPC, SD, and Lipobean^® ^at 0-1 mg/ml for 24 h. Cell viabilities were measured by MTT assay (Figure 1A). PPC and Lipobean^® ^at 0.1 mg/ml already induced 3T3-L1 cell death in about 50% of the tested populations, whereas SD at 0.1 mg/ml did not show cytotoxic activity. The morphology of 3T3-L1 cells after PPC, SD, and Lipobean^® ^treatment of 0.1 mg/ml is shown in Figure 1B. After Lipobean^® ^treatment, cell membrane disturbance was evident because of cell death. However, PPC-induced cell death produced different morphological changes. We next tested the viability of normal human fibroblast cell line, CCD-25Sk, after treatment with PPC, SD, and Lipobean^®^. PPC had little influence on CCD-25Sk cell viability at the concentrations tested, whereas SD and Lipobean^® ^significantly decreased cell viability by about 80% at 1 mg/ml (Figure 1C).

### Recovery effects of media change on 3T3-L1 and CCD-25Sk cell death

**Figure 2:**
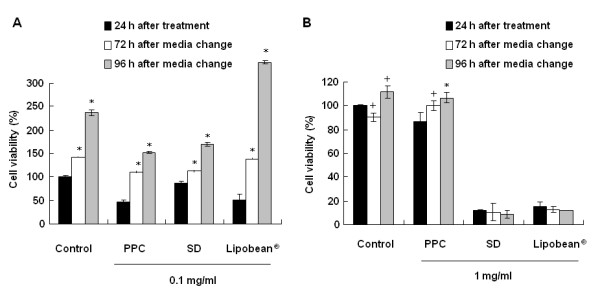
**Effects of media change on cell recovery**. (A) 3TC-L1 cells were treated with PPC, SD, Lipobean^® ^at 0.1 mg/ml. (B) CCD-25Sk cells were treated with PPC, SD, Lipobean^® ^at 1 mg/ml. After 24 h, the media was removed and then was changed with fresh media. The cell viability was measured by MTT assay after 72-96 h incubation in fresh media. The data represent the mean ± S.D. of triplicate assays expressed as percentages of the control. Each experiment was repeated independently at least twice, and the representative results are shown. + *P *< 0.05, * *P *< 0.01 compared to 24 h treated each group.

To examine the recovery from PPC, SD, or Lipobean^®^-induced cell death, 3T3-L1 and CCD-25Sk cells were supplied with fresh medium for 72 h and 96 h, after which the cells were treated with PPC, SD, and Lipobean^® ^at 0.1 and 1 mg/ml. Cell viability was then measured by the MTT assay. PPC-induced 3T3-L1 cell death was recovered in a minimal fashion, whereas Lipobean^®^-induced cell death was dramatically abrogated (Figure 2A). On the other hand, SD- or Lipobean^®^-induced CCD-25Sk cell death was not recovered by a change of fresh media, whereas the viability of PPC-treated CCD-25Sk cells was increased significantly after medium change (Figure 2B).

### PPC induces stress-regulated signaling pathway and apoptotic pathway activation

**Figure 3:**
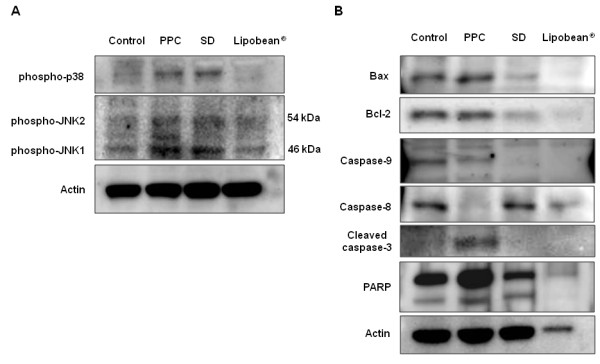
**Effects of PPC, SD, and Lipobean^® ^on apoptotic pathways**. (A) After serum starvation, 3T3-L1 pre-adipocytes were treated with PPC, SD, and Lipobean^® ^at 0.1 mg/ml for 4 h. Whole cell lysates were then subjected to Western blot analysis with antibodies against phospho-specific p38 MAPK and phospho-specific JNK. (B) After serum starvation, 3T3-L1 pre-adipocytes were treated with PPC, SD, and Lipobean^® ^at 1 mg/ml for 24 h. Whole cell lysates were then subjected to Western blot analysis with antibodies against Bax, Bcl-2, caspase-9, caspase-8, cleaved caspase-3, and PARP. Equal protein loadings were confirmed using anti-actin antibody.

Next, it was investigated whether p38 MAPK or JNK could be activated by PPC, SD, and Lipobean^® ^treatment. PPC or SD (0.1 mg/ml) resulted in the activation of p38 MAPK and JNK, while Lipobean^® ^did not (Figure 3A). We further examined changes of the apoptotic pathways induced by PPC, SD, and Lipobean^® ^(Figure 3B). PPC clearly up-regulated Bax expression in 3T3-L1 cells, but had little influence on Bcl-2 expression. Thus, PPC treatment substantially increased the Bax/Bcl-2 ratio. Caspases become active when they are cleaved into processed fragments. In this experiment, we used antibodies directed against the precursor forms of caspase-9 and caspase-8. As shown in Figure 3B, PPC cleaved and activated caspase-9 and caspase-8. An antibody directed against the cleaved form of caspase-3 was also used, and the active form of caspase-3 increased upon PPC treatment. Caspase-3 is believed to be the most efficient PARP-cleaving caspase. Accordingly, we found that the full-length PARP was cleaved after PCC treatment. However, SD and Lipobean^® ^did not show these apoptotic changes.

### Effects of PPC, SD, and Lipobean^® ^on differentiated 3T3-L1 cells

**Figure 4:**
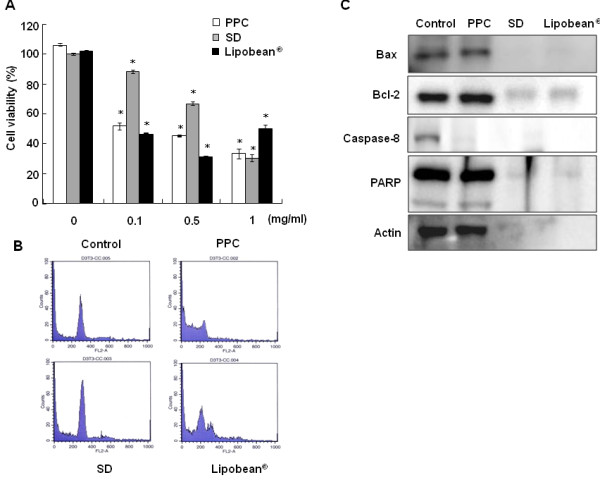
**Effects of PPC, SD, and Lipobean^® ^on differentiated 3T3-L1 cells**. 3T3-L1 pre-adipocytes were differentiated for 6 days with MDI media. (A) Differentiated 3T3-L1 cells were treated with PPC, SD, and Lipobean^® ^at 0-1 mg/ml. After 24 h, cell viability was measured by the MTT assay. The data represent the mean ± S.D. of triplicate assays expressed as percentages of the control. * *P *< 0.01 compared to the untreated control (B) Differentiated 3T3-L1 cells were treated with PPC, SD, and Lipobean^® ^at 0.1 mg/ml for 24 h. Cell cycle analysis of cells was performed by a flow cytometer. (C) Differentiated 3T3-L1 cells were treated with PPC, SD, and Lipobean^® ^at 1 mg/ml for 24 h. Whole cell lysates were then subjected to Western blot anaysis with antibodies against Bax, Bcl-2, caspase-8, and PARP. Equal protein loadings were confirmed using anti-actin antibody.

3T3-L1 pre-adipocytes were differentiated with MDI medium for 6 days. Thereafter, differentiated 3T3-L1 cell viabilities were also tested after the treatment of PPC, SD, and Lipobean^® ^at 0-1 mg/ml for 24 h (Figure 4A). The results concerning differentiated 3T3-L1 cells showed almost same tendency in 3T3-L1 pre-adipocytes. Furthermore, cell cycle changes were analyzed by flow cytometry (Figure 4B). Consistent with the cell viability results, cell cycle disruption was observed when differentiated 3T3-L1 cells were treated with PPC and Lipobean^® ^at 0.1 mg/ml. At a concentration of 1 mg/ml, we could not detect cells after SD and Lipobean^® ^treatment, because they resulted in cell membrane lysis (data not shown). For the same reason, the cell membrane was disrupted, so protein was not detected in Western blot analysis when differentiated 3T3-L1 cells were treated with 1 mg/ml of SD or Lipobean^® ^(Figure 4C). However, PPC treatment showed clear caspase-8 and PARP cleavage, indicating that PPC induced apoptosis of differentiated 3T3-L1 cells.

## Discussion

In past years, nonsurgical lipolysis has increased [7], and is the most economical use of subcutaneous injections to reduce local fat via chemical lipolysis [8]. PPC injections using a variety of PPC formulations are popular as a treatment of local adiposities [24]. However, no product intended for lipodissolution has yet been approved by the Food and Drug Administration, Medicines and Healthcare Products Regulatory Agency (MHRA), because of lack of data concerning safety and efficacy [24].

A PPC formulation generally used clinically consists of PPC and SD [24]. It has been suggested that SD, the detergent in the PPC formulation, is the active lipodissolving substance, and not PPC [25]. The main side effects of injection of PPC formulations are relatively minor, and include swelling, bruising, and sensitivity to touch under the skin in areas with the lipodissolution treatments [26]. However, since injection of SD causes focal necrosis and inflammation in human lipomas [25], it is entirely conceivable that the aforementioned side effects of PPC formulations are due to SD, not to PPC itself. Presently, we confirmed that SD induces cell membrane lysis, resulting in necrosis. Interestingly, however, the results of the MTT assay clearly indicate that PPC alone without SD induced cell death of 3T3-L1 pre-adipocytes. The results also demonstrated that the effects of inducing 3T3-L1 cell death by PPC formulation did not depend only on SD. In addition, our flow cytometric experiments showed that Lipobean^® ^treatment resulted in cell membrane lyses because of SD (data not shown). A large discrepancy in the extent of cell viability was presently evident between 0.5 mg/ml and 1 mg/ml Lipobean^® ^(Figure 1C). These Lipobean^® ^concentrations may be at the threshold for membrane stability.

In agreement with our study, PPC formulation and SD induce the lysis of various cell types including pre-adipocytes, normal human fibroblasts, endothelial cells, and skeletal muscle cells in a nonspecific manner [7]. These results are very important because the injection of PPC formulation may cause tissue necrosis. However, in our study, PPC alone did not induce cell death of CCD-25Sk normal fibroblasts. It is very important that PPC reduced only adipocyte viability specifically, but did not decrease cell viability of other cell types such as fibroblasts. On the other hand, PPC formulation and SD induced cell death of both cell types. These results indicate that the SD constituent of PPC formulations initiates the cell lysis, leading to cell death. Clearly, more study is required to confirm this suggestion.

PPC induces apoptosis in cells such as colon cancer cells [27], vascular endothelial cells [28], and macrophages [29]. Phosphorylation of stress-activated protein kinase pathways [30] and cleavage of caspases are main pathways of apoptosis [31]. Especially, the death-receptor inducing activation of caspase-8 and mitochondrial pathways is the key point of apoptotic pathways [32]. In the present study, PPC induced not only the activation of p38 and JNK, but also the cleavage of caspase-8, -9, -3, and PARP in 3T3-L1 pre-adipocytes (Figure 3). Specifically, PPC treatment unequivocably activated caspase-8 in both 3T3-L1 pre-adipocytes and differentiated 3T3-L1 cells (Figures 3B and 4C). The caspase-8 pathway is intimately involved in CD95-mediated apoptotic cell death [33,34]. These results indicate that PPC-induced apoptosis may be due to increased death receptor activations. This possibility should be further examined to elucidate PPC-induced apoptotic pathways.

Furthermore, the increase of sub-G_1 _fraction is an indication of apoptosis that naturally occurs in cells [35], and the sub-G_1 _phase in the cell cycle is increased in apoptotic cell death [36]. Therefore, to characterize apoptotic cells upon treatment of PPC, SD, and PPC formulation, flow cytometric analysis was presently performed. Treatment with 0.1 mg/ml PPC treatment increased sub-G_1 _phase in 3T3-L1 adipocytes. These results favor the view that PPC induces apoptosis of 3T3-L1 cells.

## Conclusions

In conclusion, it can be suggested that PPC alone has an apoptotic effect on 3T3-L1 adipocytes. Thus, PPC without SD should be used for the potential drug of treatment of local fat to avoid the main side effects of PPC formulation. For this purpose, a new drug delivery method will be needed for a PPC formulation without SD. Because PPC may form liposomal vesicles in aqueous solution, PPC liposomes could be adequate for a new drug delivery method.

## Abbreviations

JNK: c-Jun N-terminal kinase; MAPK: mitogen-activated protein kinase; MDI: 3-isobutyl-1-methylxanthine: dexamethasone: insulin; PARP: poly(ADP-ribose) polymerase; PPC: phosphatidylcholine; SD: sodium deoxycholate.

## Competing interests

The authors declare that they have no competing interests.

## Authors' contributions

HL participated in data acquisition, interpretation, and the writing of this manuscript. JHL, SYK, HYY, KJB, NSK, YY, and JHJ participated in the study design and data interpretation. DSK contributed to the experimental design, data interpretation, editing, and submission of this manuscript. All authors read and approved the final manuscript.
